# Calprotectin Is a Useful Tool in Distinguishing Coexisting Irritable Bowel-Like Symptoms from That of Occult Inflammation among Inflammatory Bowel Disease Patients in Remission

**DOI:** 10.1155/2013/620707

**Published:** 2013-02-06

**Authors:** Lars-Petter Jelsness-Jørgensen, Tomm Bernklev, Bjørn Moum

**Affiliations:** ^1^Department of Gastroenterology, Østfold Hospital Trust, 1603 Fredrikstad, Norway; ^2^Department of Health Sciences, Østfold University College, K.G. Meldahlsvei 9, 1671 Kråkerøy, Norway; ^3^Department of Research, Telemark Hospital Trust, 3710 Skien, Norway; ^4^Institute of Clinical Medicine, University of Oslo-Oslo University Hospital, 0450 Oslo, Norway

## Abstract

*Background and Aim*. In the inflammatory bowel diseases (IBDs), many symptoms are similar to the functional disorder irritable bowel syndrome (IBS). A challenge is thus to distinguish symptoms of IBD from IBS. The aim of this study was to investigate the levels of calprotectin in IBS-positive IBD patients in remission. *Methods*. Remission was defined as a simple clinical colitis activity index (SCCAI) or simple crohn's disease activity index (SCDAI) score of less than three and less than four, respectively. The Rome II criteria were used to identify cases, and the calprotectin ELISA test was used to quantify calprotectin in stools. *Results*. The Rome II criteria were fulfilled in 24.6% of ulcerative colitis (UC) patients, while the comparable number for Crohn's disease (CD) was 21.4%. There was a tendency for elevated fecal calprotectin levels in IBS-positive patients, regardless of diagnosis. However, these differences were only significant in CD. *Conclusions*. Calprotectin levels are elevated in subgroups of IBD patients that are in remission and have coexisting IBS-like symptoms. This study underscores the clinical usefulness of a noninvasive marker to distinguish patients in need of intensified followup from those that do not need further workup.

## 1. Introduction

Both ulcerative colitis (UC) and Crohn's disease are chronic gastrointestinal disorders, which are often referred to as inflammatory bowel disease (IBD). IBD is characterised by a pattern of symptoms that most commonly alternate between periods of remission and exacerbation [1]. Since IBD shares many common features with the functional gastrointestinal disorder irritable bowel syndrome (IBS), a usual clinical challenge is to distinguish symptoms of IBD from that of IBS [2, 3]. Among IBD patients deemed by conventional tests to be in remission, this is particularly challenging. Simrén et al. [4] found that 57% of patients with Crohn's disease (CD) and 33% of patients with ulcerative colitis (UC) have IBS-like symptoms, despite the long-standing remission. Recently, Keohane et al. [3] confirmed the findings of Simrén et al. [4] and reported the prevalence of IBS-like symptoms among CD and UC to be 59.7% and 38.6%, respectively. 

Since IBS and IBD, in many cases, cannot be separated from each other on the basis of symptoms, an objective marker would be preferable. Even though the serological panel for IBD has been expanding in recent years, the value of such parameters in distinguishing IBD from IBS has not been successful [2]. On the other hand, Roseth et al. [5], Schoepfer et al. [2], and Keohane et al. [3] demonstrated promising results when measuring the zinc-binding protein calprotectin in feces. Interestingly, IBS-positive patients were found to have significantly higher levels of calprotectin [2, 3].

The aim of this study was to investigate the levels of calprotectin in IBS-positive IBD patients in clinical remission.

## 2. Materials and Methods

### 2.1. Subjects

Patients who were over the age of 18 years, had IBD that was previously verified clinically, endoscopically, and histologically, and were either in remission or with mild to moderate disease activity, defined as SCCAI (simple clinical colitis activity index) [6] or SCDAI (simple Crohn's disease activity index) [7] score less than 10, were eligible for inclusion in this study. Patients were excluded if they had cognitive impairment, and they were judged to be unlikely to comply with the study procedures, or if they had already participated in other studies. Clinical remission was defined as an SCCAI or SCDAI score of less than three and less than four in UC and CD, respectively. Patients were consecutively recruited from three outpatient clinics in the southeast of Norway during routine followup. At each of the centres, a senior gastroenterologist was in charge of the study performance. 

### 2.2. Clinical and Sociodemographic Data

Sociodemographic variables were gathered by way of interview. Data regarding clinical status and symptoms were obtained from medical records, laboratory tests, and disease activity indices (SCCAI/SCDAI) [6, 7]. The Rome II criteria for IBS were used to define IBS cases [8] (Table 1). 

### 2.3. Fecal Calprotectin

The calprotectin ELISA test (ALP) (Calpro AS, Oslo, Norway) was used to quantify the level of calprotectin in a stool sample obtained from each patient. The test is previously validated and has also been approved by the Food and Drug Administration (FDA) as a measurement tool to distinguish IBD from IBS. All procedures were followed according to the recommendations made by the manufacturer [9].

### 2.4. Statistical Analysis

To assess the characteristics of the sample, we used descriptive analysis, frequencies, and the *χ* ^2^ test. *t*-tests were used to evaluate the differences in parametric data.

All tests were 2 sided and with a 5% significance level. All statistics were performed by the use of the Predictive Analytics Software, PASW, version 18.0 (IBM Corporation, Route 100, Somers, NY, USA).

## 3. Results

One hundred and forty-four patients diagnosed with either UC or CD gave written informed consent for participation in the study. When a cutoff of <4 on the SCDAI and <3 on the SCCAI was used to define clinical remission, and patients on current steroid treatment had been excluded, a total of 89 patients, 63.6% of the initial sample, were identified. Out of these 89 patients, 61 were diagnosed with UC and 28 with CD.

### 3.1. Fecal Calprotectin in Patients with and without IBS-Like Symptoms

The Rome II criteria were fulfilled in 24.6% of UC patients, while the comparable number for CD was 21.4%. Primary characteristics of patients are presented in Table 1. As shown in this table, there was a tendency for elevated fecal calprotectin levels in IBS-positive patients, regardless of diagnosis. However, these differences were only significant in CD. 

When using a score of <100 **μ**g/g calprotectin as a cutoff value for an endoscopically inactive disease, as suggested by af Björkesten et al. [10], 9 IBS-positive patients had calprotectin values >100 **μ**g/g, with a mean value of 808.2 and 872.7 **µ**g/g in UC and CD, respectively. The comparable number in IBS-positive patients with calprotectin values less than 100 **µ**g/g was 40.5 and 5.3 (Table 2).

## 4. Discussion

In this study, we investigated the prevalence of irritable-bowel-syndrome-(IBS-) like symptoms among inflammatory bowel disease (IBD) patients deemed to be in clinical remission. We found the prevalence of patients fulfilling the Rome II criteria for IBS-like symptoms to be somewhat lower than previously reported [3, 4, 11]. The reason for this is unclear, but potential explanations might be found in factors not taken into account, such as the duration of remission. Minderhoud et al. [11] reported, using the Rome II criteria, that the prevalence of IBS-like symptoms among IBD patients in remission was 31.5% and 41.7% for UC and CD, respectively. However, some differences were noted when the same cohort of patients was reevaluated using the Manning criteria, where the number of cases in CD was nearly halved [11]. Consequently the prevalence and detection of such symptoms are dependent on which criteria and cutoff value are used.

Since IBS is symptomatically similar to IBD, it is particularly difficult to distinguish the two from each other, without having to investigate patients endoscopically. In addition, there is nothing more frustrating from the patients' point of view than to be told that they have IBS symptoms when the patients are convinced that it is the active the IBD that is the cause of their problems. From a clinical perspective, it is therefore preferable to “ruleout” the IBS-like symptoms by the use of a noninvasive marker, for example, calprotectin, in order to ensure whether further IBD workup or intensified treatment is justified. Another key aspect is the inability of clinical activity indices, such as the SCCAI and SCDAI, to indicate clinical remission alone. Even though the CDAI indicates remission, endoscopic findings have proven otherwise [10]. Therefore, bowel symptoms in these patients do not need to necessarily be IBS. Even though we defined cutoff values for clinical remission at <3 and <4 on the SCCAI and SCDAI, respectively, some patients had calprotectin values indicating ongoing inflammatory activity. Keohane et al. [3] found a significantly elevated level of calprotectin in both IBS-positive UC and CD patients, and Schoepfer et al. [2] argued that the fecal test was highly accurate in discriminating IBD from IBS. However, we agree with Keohane et al. [3] that—unless otherwise proven—symptoms of IBD should be regarded as IBD and not as IBS-like symptoms. The latter is clearly underscored by the level of calprotectin among IBS-positive patients [3]. Even though we were not able to replicate the findings of Keohane et al. [3] in UC, merely in CD, there was a clear tendency for elevated calprotectin levels in IBS-positive patients, regardless of diagnosis. 

As shown in the present study and the study from Ireland [3], it is important to combine clinical activity indices with a noninvasive marker, such as calprotectin. The use of laboratory markers is, in this sense, not an unnecessary but rather a useful tool [12]. On the other hand, several different cut-off levels of calprotectin have been proposed both in order to distinguish IBD from IBS and to indicate remission. Tibble et al. [13] reported that 30 **μ**g/g had 100% sensitivity in discriminating IBS from IBD. Others like D'Incà et al. [14] and Sipponen et al. [15] have found 130 and 200 **μ**g/g, respectively, to be potential useful cutoff values for remission and activity. In a recently published paper by Sipponen's group [10], the best cutoff value for endoscopic remission was found to be 94 **μ**g/g. Interestingly, when setting a cutoff level of calprotectin at <100 **μ**g/g—in accordance with the findings of Björkesten and collegues [10]—we found significantly different mean levels for IBD patients with IBS-like symptoms above and under the cutoff limit. The important message is that even though patients are judged to be in clinical remission, they may not be so. Calprotectin cutoff levels may therefore be helpful in distinguishing those patients that most likely have coexisting IBS-like symptoms and those patients that need more workup or intensified treatment. 

Therefore, in conclusion, calprotectin levels are elevated in subgroups of IBD patients that are in clinical remission and have coexisting IBS-like symptoms, indicating subclinical activity. This study underscores the clinical usefulness of a noninvasive marker, such as calprotectin, to distinguish IBD patients in need of intensified followup from those that potentially do not need further workup.

## Figures and Tables

**Table 1:** Sociodemographic and clinical data among IBD patients with or without IBS.

		UC			CD	
	IBS negative	IBS positive	*P* value	IBS negative	IBS positive	*P* value
	*n* = 46	*n* = 15		*n* = 22	*n* = 6	
Age—mean (SD)	50.2 (15.6)	38.7 (10.7)	0.01	38.9 (16.3)	30.5 (8.6)	Ns
Gender:						
% female	43	33	Ns	73	100	Ns
Disease duration—mean (SD)	9.3 (10.7)	6.8 (4.9)	Ns	6.2 (5.8)	8.8 (8.6)	Ns
Calprotectin	189.3 (348.7)	347.7 (443.9)	Ns	156.6 (193.3)	439.0 (508.7)	0.04
Smoking:						
% yes	26	27	Ns	32	17	Ns
Employment:						
% yes	61	73	Ns	68	83	Ns

IBD: inflammatory bowel disease, IBS: irritable bowel syndrome, UC: ulcerative colitis, CD: Crohn's disease, SD: standard deviation, and Ns: no significance.

**Table 2:** Mean (SD) levels of calprotectin in IBD patients with or without IBS-like symptoms using a cutoff of <100 *μ*g/g.

Calprotectin	IBS positive	IBS negative	*P* value
	Ulcerative colitis		

<100 *μ*g/g	*n* = 9	*n* = 27	0.14
	40.5 (36.9)	23.7 (26.4)	
≥100 *μ*g/g	*n* = 6	*n* = 12	0.26
	808.3 (353.6)	562 (446.8)	

	Crohn's disease		

<100 *μ*g/g	*n* = 3	*n* = 9	0.17
	5.3 (4.6)	19.4 (16.0)	
≥100 *μ*g/g	*n* = 3	*n* = 9	<0.01
	872.7 (287.7)	293.7 (191.9)	

SD: standard deviation, IBD: inflammatory bowel disease, and IBS: irritable bowel syndrome.
